# Cytotoxicity and sub-acute toxicity in Crl:CD (SD) rats of traditional herbal formula Ojeok-san

**DOI:** 10.1186/s12906-015-0582-y

**Published:** 2015-03-01

**Authors:** Soo-Jin Jeong, Jung-Im Huh, Hyeun-Kyoo Shin

**Affiliations:** Herbal Medicine Formulation Research Group, Korea Institute of Oriental Medicine, 1672 Yuseong-daero, Yuseong-gu, Daejeon, Republic of Korea; Division of Non-clinical Studies, Korea Institute of Toxicology, PO Box 123, 100 Jangdong, Yusung-gu, Daejeon, 305-343 Republic of Korea

**Keywords:** Ojeok-san, Herbal formula, Cytotoxicity, Sub-acute toxicity

## Abstract

**Background:**

Although Ojeok-san (OJS), an oriental herbal formula, has been used in Asian countries including Korea, China and Japan to treat the common cold and illnesses including fatigue and gastrointestinal disorders, there is little information of its safety and toxicity *in vivo* and *in vitro*.

**Methods:**

In the present study, we investigated oral toxicity of OJS over 4 weeks through repeated administration to Crl:CD (SD) rats and its cytotoxicity against various cells as a part of safety evaluation. Animals were given a daily gavage treatment of OJS in daily dosages of 0, 500, 1000 or 2000 mg/kg for 4 weeks. Cytotoxicity assay was conducted at various concentrations in 23 different cell lines including neuroblastoma, glioblastoma, hepatocarcinoma, melanoma, leukemia, colon cancer, breast cancer, keratinocytes, phechromocytoma, prostate cancer, bronchial epithelial cells, and gastric adenocarcinoma.

**Results:**

OJS did not induce significant changes in mortality, food consumption, organ weights, hematology, serum biochemistry, and urinalysis, except for decrease in number of white blood cells over 1000 mg/kg/day female group. Thus, the no observed adverse effect level (NOAEL) is more than 2000 mg/kg/day for male and 500 mg/kg/day for female rats. In addition, OJS had no cytotoxicity against all tested cells.

**Conclusions:**

Collectively, our data indicate that OJS may be a safe drug although additional studies in the near future will be required before clinical trials can be taken.

## Backgrounds

Drug safety is one of major issue in new drug development. The Food and Drug Administration (FDA) in most countries provides guidance on drug safety information for developing and disseminating new drugs with no or less toxicity as well as side effect. With the development of traditional medicine, concerns on the safety and potential toxicity of herbal medicines are increasing. However, there is little information of herbal medicine on the safety compared with synthetic drugs.

Ojeok-san (OJS; *wuji-san* in China and *goshaku-san* in Japan) consisting of 17 medicinal herbs has long been used for treating common cold and illnesses including fatigue and gastrointestinal disorders in Asian countries including Korea, China and Japan. According to statistical data of the Korea National Health Insurance Corporation in 2011, OJS is the most frequently used herbal formulas of Oriental health treatments by 56 prescriptions. OJS is occupied 23% of the days of medication and 30% of medical expenses in Korea [1].

In experimental studies, OJS has been reported to have several biochemical activities such as anti-cancer [2], anti-inflammation [3,4], anti-hyperglycemia [5], anti-clastogenic effect [6] and immune regulation [7]. In addition, Kim et al. recently established OJS administration criteria and a questionnaire to evaluate its holistic effects on patients with low back pain (LBP) [8]. Our group reported toxicological data on acute and sub-chronic toxicity [9] as well as genotoxicity [10] of OJS in Sprague–Dawley (SD) rats. However, sub-acute toxicity information is also required to establish the safety and efficacy of OJS.

Therefore, we here assessed the toxicity of a 4-week repeated oral doses of OJS in Crl:CD (SD) rat model according to guidelines established by the Organization for Economic Cooperation and Development (OECD) for the testing of chemicals in accordance with the current regulations for Good Laboratory Practice Regulations [11]. Our data demonstrate that OJS orally administered to rats is safe without drug-related toxicity in 4-week repeated administration study. Furthermore, the cytotoxicity of OJS was evaluated against various cell lines from different origins.

## Methods

### Preparation of Ojeok-san (OJS)

The OJS consisting of seventeen herbal medicines (Table 1) and each crude drug was purchased from Kwangmyoungdang (Ulsan, Korea). The origin of materials was confirmed taxonomically by Profs. Je-Hyun Lee (Dongguk University, Gyeongju, Korea) and Young-Bae Seo (Daejeon University, Daejeon, Korea). A decoction of OJS was prepared as previously described [9]. In brief, a mixture of chopped crude herbs was extracted in 10 volume of distilled water at 100°C for 2 h in an herb extractor (COSMOS-660, Kyungseo Machine Co., Incheon, Korea). The solution was filtered and lyophilized with a freeze drier (PVTFD100R, Ilshin Lab. Ltd., Korea) (The yield = 11.3%). The HPLC pattern of OJS extract was identified for quality control [9].

**Table 1:** **The composition of OJS**

**Latin name**	**Amount (g)**	**Company of purchase**	**Source**
Atractylodis Rhizoma	7.50	Kwangmyoungdang	China
Ephedrae Herba	3.75	Kwangmyoungdang	China
Citri Unshius Pericarpium	3.75	Kwangmyoungdang	Jeju, Korea
Magnoliae Cortex	3.00	Kwangmyoungdang	China
Platycodonis Radix	3.00	Kwangmyoungdang	Muju, Korea
Aurantii Fructus Immaturus	3.00	Kwangmyoungdang	China
Angelicae Gigantis Radix	3.00	Kwangmyoungdang	Bonghwa, Korea
Zingiberis Rhizoma	3.00	Kwangmyoungdang	Taean, Korea
Paeoniae Radix	3.00	Kwangmyoungdang	Euiseong, Korea
Poria Sclerotium	3.00	Kwangmyoungdang	Pyeongchang, Korea
Angelicae Dahuricad Radix	2.63	Kwangmyoungdang	Uljin, Korea
Cnidii Rhizoma	2.63	Kwangmyoungdang	Yeongyang, Korea
Pinelliae Tuber	2.63	Kwangmyoungdang	China
Cinnamomi Cortex	2.63	Kwangmyoungdang	Vietnam
Glycyrrhizae Radix et Rhizoma	2.25	Kwangmyoungdang	China
Zingiberis Rhizoma Crudus	3.75	Kwangmyoungdang	Yeongcheon, Korea
Allii Fistulosi Bulbus	3.75	Kwangmyoungdang	Hanam, Korea
Total amount	56.25		

### Animals

The animal studies were conducted according to the guidance of the Institutional Animal Care and Use Committee in Korea Institute of Toxicology (KRICT) (accredited by AAALAC International, 1998) under the Good Laboratory Practice Regulations for Non-clinical Laboratory Studies and approved by Korea Institute of Oriental Medicine Institutional Animal Care and Use Committee (Daejeon, Korea).

Specific pathogen-free Crl:CD (SD) rats (n = 20/gender) were obtained from the Orient Bio Co. (Seoul, Korea) and used after 2 weeks of quarantine and acclimatization. The animals were housed in a room maintained at 22 ± 3°C under a relative humidity of 50 ± 20% with artificial lighting from 08:00 to 20:00 and 12–15 air changes per hour. The animals were kept in stainless-steel wire-mesh cages and allowed sterilized tap water and commercial rodent chow (PMI Nutrition International, Richmond, VA, USA) ad libitum.

### Group assignment and treatment

Healthy male and female rats were assigned to four groups (5 male and 5 female/group) using Path/Tox System 4.2.2 (Xybion Medical Systems Corporation, Cedar Knolls, NJ, USA). OJS extract was dissolved in distilled water for injection (Choong-wae Pharmaceutical, Ltd., Seoul, Korea) and administered by oral gavage at doses of 0, 500, 1000 or 2000 mg/kg once a day for 4 weeks. Distilled water was given to the animals as the vehicle control. The daily dose (10 ml/kg body weight) of OJS was calculated based on the most recently recorded body weights of individual animals.

### General observations

Clinical signs and mortality were recorded twice a day (before and after treatment) throughout the study period. All clinical signs were recorded individually for type, observation day/time and duration using Path/Tox System 4.2.2 (Xybion Medical Systems Corporation). The body weight of each rat was measured at the initiation of treatment and once a week during the study period. Food consumption was measured at the start of treatment and weekly throughout. Daily food consumption was determined by measuring the weight of chow supplied and remaining in metabolic cages each day. External eye examination was carried out during the last week of treatment with an indirect binocular ophthalmoscope (IO-H, Neitz Instrument Co., Tokyo, Japan), and the appearance of the conjunctiva, sclera, cornea, lens, and iris of each eye was recorded.

### Urinalysis, hematology and serum biochemistry

During the last week of treatment, urinalysis was conducted on samples collected overnight in metabolic cages using a Multistix 10 SG (Bayer, Whippany, NJ, USA) and urine chemical analyzer (Clinitek-500, Ramsey, MN, USA). Analysis included urine volume, glucose, bilirubin, ketone body, specific gravity, occult blood, pH, urobilinogen, and color.

Animals were fasted overnight prior to blood collection or necropsy. Blood was drawn from the posterior vena cava under isoflurane anesthesia. Samples were collected in CBC bottles containing EDTA-2 K (Sewon Medical Co., Cheonan, Korea), and were analyzed to determine red blood cell count (RBC), white blood cell count (WBC), differential WBC count, hemoglobin concentration (HGC), hematocrit (HCT), mean corpuscular volume (MCV), mean corpuscular hemoglobin (MCH), mean corpuscular hemoglobin concentration (MCHC), platelet (PLT) and reticulocyte (RET) using an ADVIA120 Hematology System (Bayer). Prothrombin time (PT) and activated partial thromboplastin time (APTT) were determined in blood samples treated with 3.2% sodium citrate using a coagulometer (ACL 300 plus, Instrumentation Laboratory, Milan, Italy).

For serum biochemistry, blood samples were centrifuged at 3000 rpm for 10 min and analyzed with an autoanalyzer (Toshiba 200FR NEO, Toshiba Co., Tokyo, Japan). The analysis included alanine aminotransferase (ALT), aspartate aminotransferase (AST), alkaline phosphatase (ALP), gamma glutamyl transpeptidase (GGT), blood urea nitrogen (BUN), creatinine (CREA), creatine kinase (CK), glucose (GLU), total cholesterol (TCHO), albumin (ALB), albumin/globulin ratio (A/G), total protein (TP), triglyceride (TG), total bilirubin (TBIL), phospholipids (PL), sodium (Na), potassium (K), calcium (Ca), chloride (Cl), and inorganic phosphorus (IP).

### Necropsy

All surviving animals were anesthetized with isofluorane and sacrificed by aortic exsanguination prior to necropsy. Complete gross postmortem examinations were performed on all animals. Absolute organ weights were measured and relative organ weights (organ-to-body weight ratios) were calculated for the following organs: brain, pituitary gland, adrenal gland, liver, spleen, kidneys, heart, thymus, lung, salivary gland, thyroids, testes, ovaries, epididymides, seminal vesicle, prostate, and uterus.

### Cytotoxicity assay

Twenty-three of various cell lines were obtained from the American Type Culture Collection (ATCC, Rockville, MD, USA) or Korean Cell Line Bank (Seoul, Korea). Cytotoxic effects of OJS against the cell lines were measured by 3-(4,5-dimethylthiazol-2-yl)-2,5-diphenyl tetrazolium bromide (MTT) assay as previously described [12]. Cell viability was calculated as a percentage of viable cells in drug-treated group *versus* untreated control by following equation.

$$ \mathrm{Cell}\ \mathrm{viability} = \left[\mathrm{O}\mathrm{D}\ \left(\mathrm{O}\mathrm{J}\mathrm{S}\right)-\mathrm{O}\mathrm{D}\ \left(\mathrm{Blank}\right)\right]/\left[\mathrm{O}\mathrm{D}\ \left(\mathrm{Control}\right) - \mathrm{O}\mathrm{D}\ \left(\mathrm{Blank}\right)\right] \times 100 $$

### Statistical analysis

Data collected during the study were examined for the variance homogeneity using Bartlett’s test. When Bartlett’s test indicated no significant deviation from the variance homogeneity, a one-way ANOVA was performed at α = 0.05. When significance was noted, a multiple comparison test (Dunnett’s test) was conducted to determine which pairs of groups were significantly different. Where significant deviations from variance homogeneity were observed, a nonparametric comparison test (Kruskal–Wallis test) was conducted. When a significant difference was observed in the Kruskal–Wallis test, the Dunn’s Rank Sum test was conducted to determine the specific pairs. Statistical analyses were performed using the Path/Tox System (ver. 4.2.2). The level of significance was taken as *p* < 0.05 or 0.01.

## Results and discussion

Recently, the use of herbal medicines is increased and thus the safety information is considered an important issue for herbal prescription. U.S. FDA has restricted the use of products containing aristolochic acid including botanical products marketed as traditional medicine since they can increase to occur in renal failure and renal cancer. It is of note that 11 kinds of herbal formulas are included in the restricted product list. Thus, safety report of medicinal herb or herbal formula is crucial step for their drug development and medication counseling. We previously reported that no mortality and no abnormality in clinical signs, body weight, and necropsy findings for any of the animals in the acute and sub-chronic toxicity study following oral administration of OJS, a Korean traditional herbal formula [9]. In the present study, we evaluated safety of OJS by *in vivo* sub-acute toxicological analysis and *in vitro* cytotoxicity test.

### Body weight, organ weight and food intake changes in SD rats treated with OJS extract for 4 weeks

**Figure 1:**
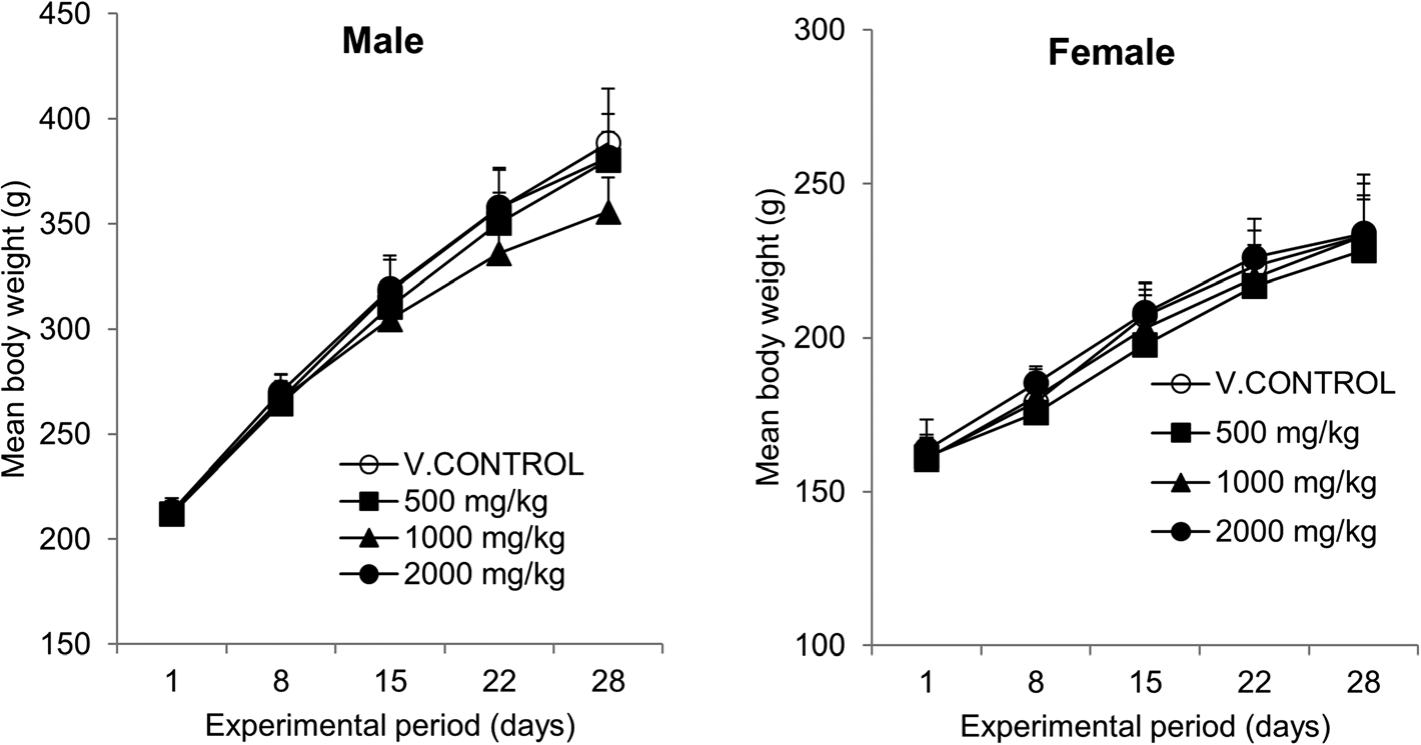
**Mean body weight changes of male (left panel) and female (right panel) rats treated with OJS at doses of 0 (○), 500 (■), 1,000 (▲), and 2,000 (●) mg/kg/day for 4 weeks.** Values are presented as mean ± SD.

Sub-acute toxicity of OJS extract was assessed in male or female Crl:CD (SD) rats (n = 24/gender) in accordance with OECD guideline. OJS extract was orally administered for 4 weeks at 500, 1000 or 2000 mg/kg/day. Body weight of male or female rats was increased in a time-dependent manner (Figure 1). However, no effect of OJS extract was observed on body weight change in both genders of rats compared with vehicle control.

**Figure 2:**
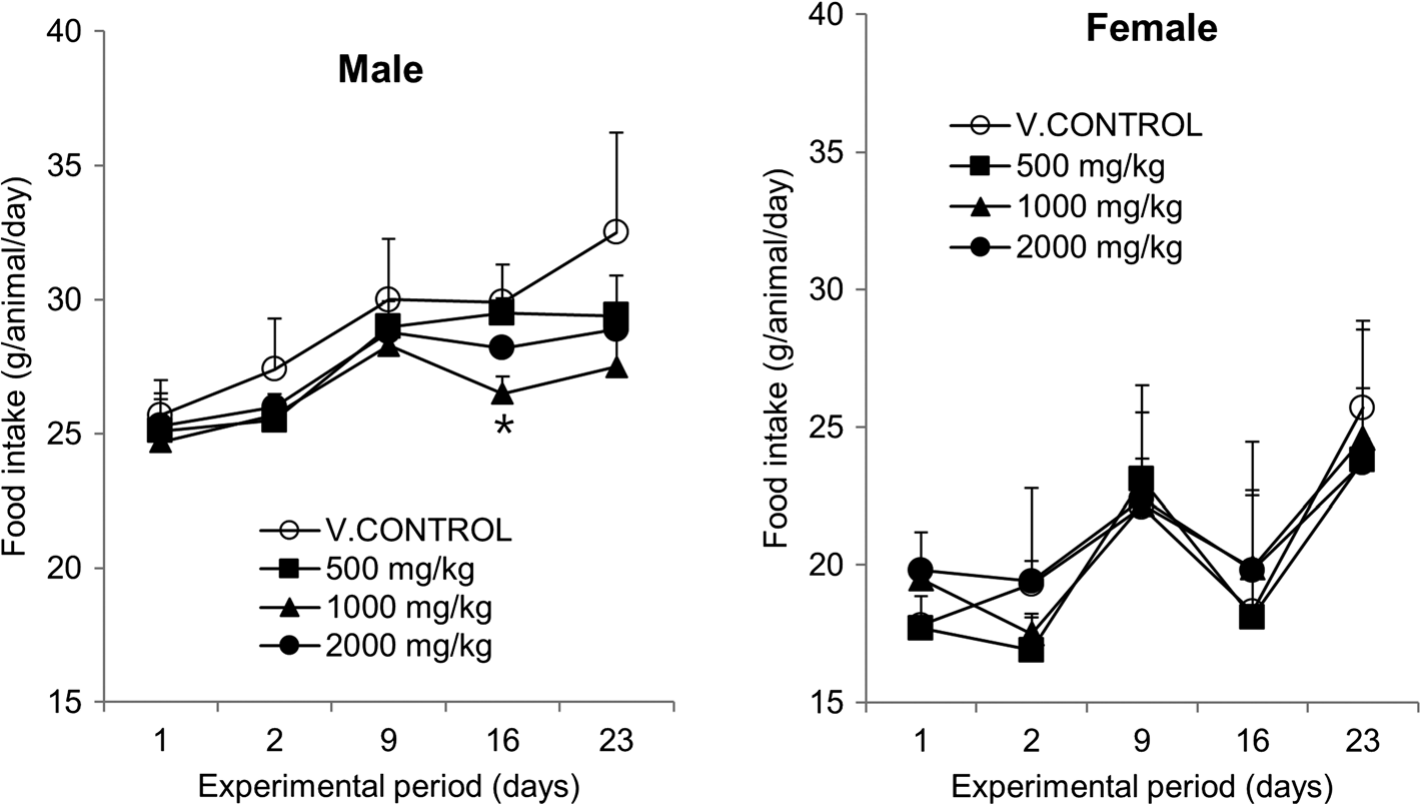
**Food intake in male (left panel) and female (right panel) rats treated with OJS at doses of 0 (○), 500 (■), 1,000 (▲), and 2,000 (●) mg/kg/day for 4 weeks.** Values are presented as mean ± SD. Significant difference, ^*^ *p* < 0.05 from V.CONTROL group.

Loss of food intake in OJS extract-treated rats was found in male group compared with vehicle control. Significant differences were observed following treatment with 1000 mg/kg at day 16. At day 23, results revealed that administration of OJS with 500, 1000 or 2000 mg/kg reduced food intake compared with vehicle control without statistical significance (Figure 2, left panel). However, we could not observe change of body weight in these groups, indicating that any alterations in food intake in male rats are regarded as incidental due to variations in measured values. In contrast, no food consumption changes were observed in female group (Figure 2, right panel).

In addition, changes in relative organ weights were also evaluated as shown in Table 2. No significant difference between control and OJS extract-treated groups was detected in terms of the relative weights of 15 different organs in both male and female animals. Furthermore, major clinical signs such as salivation and loss of fur were not observed in both male and female rats treated with OJS extract (data not shown).

**Table 2:** **Relative organ weights rats treated with OJS for 4 weeks**

**Dose (mg/kg/day)**	**0**	**500**	**1000**	**2000**
**Male**				
Brain	1.966 ± 0.0689	2.036 ± 0.0512	1.922 ± 0.0454	1.975 ± 0.0695
Pituitary gland	0.010 ± 0.0017	0.010 ± 0.0020	0.011 ± 0.0032	0.011 ± 0.0012
Liver	11.204 ± 0.7302	12.838 ± 0.7539	10.520 ± 0.9710	10.789 ± 0.6777
Spleen	0.720 ± 0.0892	0.716 ± 0.0400	0.638 ± 0.0786	0.711 ± 0.1093
Heart	1.225 ± 0.0835	1.138 ± 0.0591	1.096 ± 0.1013	1.194 ± 0.0311
Thymus	0.570 ± 0.0916	0.553 ± 0.1395	0.544 ± 0.0761	0.549 ± 0.1076
Salivary glands	0.604 ± 0.0415	0.572 ± 0.0318	0.548 ± 0.0415	0.605 ± 0.0289
Seminal vesicle	1.124 ± 0.1204	0.956 ± 0.3066	0.890 ± 0.2533	0.986 ± 0.1801
Prostate	0.432 ± 0.0821	0.371 ± 0.0599	0.445 ± 0.0568	0.464 ± 0.0810
Kidneys	2.934 ± 0.2372	3.117 ± 0.1676	2.803 ± 0.2866	2.947 ± 0.2479
Adrenal glands	0.054 ± 0.0069	0.062 ± 0.0098	0.055 ± 0.0022	0.055 ± 0.0079
Testes	3.079 ± 0.1499	3.043 ± 0.1776	2.952 ± 0.1368	2.845 ± 0.5047
Epididymides	0.896 ± 0.0475	0.880 ± 0.0445	0.840 ± 0.0445	0.862 ± 0.1289
Lung	1.357 ± 0.1520	1.473 ± 0.1236	1.322 ± 0.0734	1.377 ± 0.1054
Thyroid/Parathyroid	0.019 ± 0.0038	0.020 ± 0.0034	0.017 ± 0.0028	0.018 ± 0.0023
**Female**				
Brain	1.851 ± 0.0776	1.840 ± 0.0889	1.815 ± 0.0668	1.833 ± 0.0563
Pituitary gland	0.012 ± 0.0025	0.014 ± 0.0015	0.014 ± 0.0028	0.010 ± 0.0023
Liver	7.367 ± 0.4526	7.096 ± 0.6750	7.435 ± 0.8109	8.006 ± 0.2126
Spleen	0.540 ± 0.0879	0.499 ± 0.0512	0.483 ± 0.0537	0.474 ± 0.0788
Heart	0.807 ± 0.0278	0.762 ± 0.0647	0.820 ± 0.0831	0.833 ± 0.0316
Thymus	0.545 ± 0.1480	0.491 ± 0.0715	0.426 ± 0.0483	0.467 ± 0.0627
Salivary glands	0.412 ± 0.0221	0.387 ± 0.0280	0.403 ± 0.0293	0.410 ± 0.0266
Kidneys	1.807 ± 0.1272	1.877 ± 0.1172	1.930 ± 0.2121	1.885 ± 0.0786
Adrenal glands	0.074 ± 0.0087	0.070 ± 0.0118	0.077 ± 0.0127	0.070 ± 0.0122
Ovaries	0.084 ± 0.0104	0.076 ± 0.0070	0.093 ± 0.149	0.094 ± 0.0171
Lung	1.102 ± 0.0369	1.039 ± 0.0994	1.069 ± 0.1336	1.098 ± 0.0551
Thyroid/parathyroid	0.014 ± 0.0021	0.016 ± 0.0048	0.016 ± 0.0036	0.017 ± 0.0021
Uterus/cervix	0.451 ± 0.1435	0.409 ± 0.1121	0.576 ± 0.1598	0.458 ± 0.0742

Values are presented as mean ± SD.

### Hematology, serum biochemistry and urinalysis in SD rats treated with OJS extract for 4 weeks

In male group, there was no significant hematological change in OJS extract-treated group compared with vehicle control. In contrast, WBC number was decreased in a dosage-dependent manner and PT was slightly increased in female rats treated with OJS extract compared with control (Table 3), suggesting that decrease in WBC number is specific for female group. The change is possibly correlated with a weak reduction of absolute and relative spleen weight in female rats although there was no statistical significance from vehicle control (Table 2).

**Table 3:** **Hematological values of animals treated with OJS for 4 weeks**

**Dose (mg/kg/day)**	**0**	**500**	**1000**	**2000**
**Male**				
WBC (10^3^/μL)	10.1 ± 3.32	10.2 ± 4.00	10.3 ± 1.05	9.8 ± 1.09
Reticulocyte (%)	2.6 ± 0.57	2.5 ± 0.30	2.5 ± 0.30	2.3 ± 0.34
Neutrophils (%)	16.0 ± 5.16	12.1 ± 4.42	11.2 ± 1.92	11.0 ± 3.17
Lymphocytes (%)	77.3 ± 5.76	82.4 ± 5.65	83.1 ± 2.03	82.0 ± 3.57
Eosinophils (%)	0.9 ± 0.23	0.7 ± 0.36	0.6 ± 0.13	0.9 ± 0.26
Monocytes (%)	3.5 ± 1.11	2.6 ± 1.07	3.0 ± 0.59	3.8 ± 0.77
Basophils (%)	1.0 ± 0.38	0.9 ± 0.40	1.0 ± 0.45	0.8 ± 0.23
Large unstained cells (%)	1.3 ± 0.40	1.2 ± 0.26	1.1 ± 0.11	1.5 ± 0.27
RBC (10^6^/μL)	7.9 ± 0.27	8.0 ± 0.12	8.2 ± 0.40	8.0 ± 0.38
Hemoglobin (g/dl)	15.7 ± 0.46	16.0 ± 0.35	16.3 ± 0.71	15.6 ± 0.64
Hematocrit (%)	46.2 ± 1.07	46.4 ± 1.10	47.7 ± 1.90	46.0 ± 1.93
MCV (fL)	58.7 ± 1.88	58.0 ± 0.56	58.3 ± 1.59	57.5 ± 1.37
MCH (pg)	19.9 ± 0.41	19.9 ± 0.27	19.9 ± 0.74	19.5 ± 0.45
MCHC (g/dl)	34.0 ± 0.63	34.4 ± 0.39	34.1 ± 0.65	33.9 ± 0.13
Platelet (10^3^/μL)	1113.0 ± 124.70	1195.0 ± 197.00	1074.0 ± 129.80	1155.0 ± 65.60
**Female**				
WBC (10^3^/μL)	11.5 ± 1.36	9.4 ± 2.39	8.0 ± 1.60^*^	6.4 ± 0.93^**^
Reticulocyte (%)	2.1 ± 0.16	2.1 ± 0.16	2.0 ± 0.53	2.2 ± 0.28
Neutrophils (%)	12.3 ± 4.32	12.3 ± 4.32	9.6 ± 2.54	11.1 ± 4.56
Lymphocytes (%)	81.8 ± 4.37	84.3 ± 4.58	84.4 ± 2.22	82.8 ± 4.28
Eosinophils (%)	0.3 ± 0.24	0.7 ± 0.15	0.7 ± 0.26	0.8 ± 0.20
Monocytes (%)	2.9 ± 0.70	3.3 ± 0.69	3.0 ± 1.12	3.0 ± 1.48
Basophils (%)	1.1 ± 0.54	1.2 ± 0.31	1.1 ± 0.39	1.2 ± 0.39
Large unstained cells (%)	1.0 ± 0.22	1.2 ± 0.34	1.2 ± 0.36	1.1 ± 0.26
RBC (10^6^/μL)	8.1 ± 0.36	8.3 ± 0.26	8.0 ± 0.22	8.1 ± 0.44
Hemoglobin (g/dl)	16.2 ± 0.51	16.5 ± 0.29	15.8 ± 0.43	16.4 ± 0.42
Hematocrit (%)	46.4 ± 1.76	47.3 ± 1.09	45.9 ± 0.86	47.2 ± 2.15
MCV (fL)	57.6 ± 0.64	56.8 ± 0.79	57.7 ± 1.92	58.2 ± 1.68
MCH (pg)	20.1 ± 0.35	19.8 ± 0.37	19.8 ± 0.76	20.4 ± 0.64
MCHC (g/dl)	34.9 ± 0.61	34.9 ± 0.30	34.4 ± 0.38	34.9 ± 0.85
Platelet (10^3^/μL)	1169.0 ± 86.40	1168.0 ± 91.10	1297.0 ± 85.10	1199.0 ± 184.20

MCV, mean corpuscular volume; MCH, mean corpuscular hemoglobin; MCHC, mean corpuscular hemoglobin concentration; PT, prothrombin time.

Values are presented as mean ± SD.

^*^ and ^**^ indicates a significant difference at *p* < 0.05 and *p* < 0.01 level, respectively, when compared with the vehicle control group.

In serum analysis, we observed OJS administration altered levels of several biochemical factors such as creatinine, total protein, total cholesterol, phospholipid and chloride in male, (Table 4). OJS also decreased triglyceride level in female rats, suggesting the possible potential drug candidate for the metabolic diseases such as obesity, diabetes, hyperlipidemia, and cardiovascular diseases. However, these changes did not have dosage-dependent correlation and may result from the individual difference within normal range [13,14]. Thus, they were not considered to be OJS-mediated abnormalities. In urinalysis values, there were no significant changes found in rats of either sex among any of the OJS-treated groups for urine volume, glucose, bilirubin, ketone body, specific gravity, occult blood, pH, urobilinogen, and color when compared with the vehicle-only control in this study (Table 5).

**Table 4:** **Serum biochemical values of animals treated with OJS for 4 weeks**

**Dose (mg/kg/day)**	**0**	**500**	**1000**	**2000**
**Male**				
Glucose (mg/dL)	96.2 ± 28.22	100.1 ± 12.23	114.6 ± 15.04	93.7 ± 10.74
BUN (mg/dL)	13.1 ± 1.73	11.2 ± 2.10	12.6 ± 0.87	13.1 ± 0.61
Creatinine (mg/dL)	0.5 ± 0.03	0.5 ± 0.02^*^	0.6 ± 0.02	0.5 ± 0.03
Total protein (g/dL)	6.5 ± 0.19	6.6 ± 0.11	6.5 ± 0.09	6.2 ± 0.15^*^
Albumin (g/dL)	4.3 ± 0.11	4.3 ± 0.06	4.2 ± 0.05	4.1 ± 0.04
Albumin/globulin ratio	1.9 ± 0.14	1.8 ± 0.08	1.9 ± 0.05	2.0 ± 0.09
Total cholesterol (mg/dL)	54.0 ± 5.92	68.2 ± 7.66^*^	72.2 ± 12.56^**^	60.4 ± 4.22
Triglycerides (mg/dL)	38.6 ± 15.88	58.0 ± 19.52	47.9 ± 10.78	46.8 ± 14.78
Phospholipid (mg/dL)	88 ± 7.90	105.0 ± 5.00^*^	108.0 ± 13.70^**^	93.0 ± 5.00
AST (IU/L)	130.3 ± 21.37	111.7 ± 17.16	114.0 ± 9.78	118.7 ± 26.26
ALT (IU/L)	32.6 ± 4.59	33.3 ± 4.18	34.3 ± 4.78	38.8 ± 2.35
Total bilirubin (mg/dL)	0.2 ± 0.02	0.1 ± 0.01	0.1 ± 0.01	0.1 ± 0.00
ALP (IU/L)	452.1 ± 54.40	472.9 ± 97.97	450.7 ± 50.77	422.1 ± 31.08
Creatine kinase (IU/L)	736.0 ± 249.90	551.0 ± 204.30	587.0 ± 113.40	665.0 ± 363.60
Ca (mg/dL)	10.9 ± 0.44	11.2 ± 0.22	11.5 ± 0.18	11.2 ± 0.53
IP (mg/dL)	10.8 ± 0.96	10.9 ± 0.42	10.7 ± 0.49	11.1 ± 0.73
Na (mmol/L)	146.0 ± 1.60	146.0 ± 0.90	146.0 ± 0.80	147.0 ± 1.50
K (mmol/L)	7.47 ± 1.57	8.7 ± 0.61	8.6 ± 0.52	7.6 ± 0.84
Cl (mmol/L)	103.0 ± 1.30	100.0 ± 0.90^*^	102.0 ± 1.60	102.0 ± 0.50
GGT (IU/L)	0.8 ± 0.33	1.2 ± 0.62	0.7 ± 0.38	0.5 ± 0.44
**Female**				
Glucose (mg/dL)	94.2 ± 16.57	90.7 ± 22.27	112.5 ± 47.45	86.8 ± 27.31
BUN (mg/dL)	17.4 ± 2.46	17.7 ± 2.90	16.7 ± 2.42	18.9 ± 3.47
Creatinine (mg/dL)	0.6 ± 0.05	0.6 ± 0.03	0.6 ± 0.07	0.6 ± 0.05
Total protein (g/dL)	6.9 ± 0.43	6.9 ± 0.28	7.0 ± 0.25	7.0 ± 0.18
Albumin (g/dL)	4.5 ± 0.30	4.6 ± 0.15	4.6 ± 0.20	4.6 ± 0.11
Albumin/globulin ratio	1.9 ± 0.08	2.0 ± 0.08	1.91 ± 0.1	2.0 ± 0.11
Total cholesterol (mg/dL)	80.6 ± 13.94	72.2 ± 12.60	62.6 ± 17.73	76.0 ± 12.90
Triglycerides (mg/dL)	41.6 ± 14.10	29.4 ± 3.39	25.0 ± 1.88^*^	26.4 ± 4.80
Phospholipid (mg/dL)	141.0 ± 22.70	126.0 ± 18.50	112.0 ± 20.40	128.0 ± 17.00
AST (IU/L)	110.2 ± 12.78	112.2 ± 20.95	110.6 ± 18.88	119.6 ± 21.56
ALT (IU/L)	26.7 ± 4.12	28.3 ± 4.46	28.9 ± 4.90	29.4 ± 4.51
Total bilirubin (mg/dL)	0.1 ± 0.01	0.1 ± 0.01	0.1 ± 0.03	0.1 ± 0.02
ALP (IU/L)	238.6 ± 37.60	266.7 ± 59.82	270.0 ± 67.43	254.9 ± 38.56
Creatine kinase (IU/L)	580.0 ± 140.90	547.0 ± 169.30	408.0 ± 76.30	656.0 ± 204.00
Ca (mg/dL)	11.9 ± 0.12	11.6 ± 0.55	11.5 ± 0.39	11.2 ± 0.22
IP (mg/dL)	10.7 ± 0.65	10.2 ± 0.56	10.0 ± 0.90	10.1 ± 0.41
Na (mmol/L)	145.0 ± 0.80	145.0 ± 1.80	145.0 ± 0.50	144.0 ± 1.10
K (mmol/L)	7.9 ± 0.76	7.7 ± 0.54	7.5 ± 0.46	7.9 ± 0.79
Cl (mmol/L)	102.0 ± 1.20	103.0 ± 1.50	103.0 ± 1.10	102.0 ± 0.80
GGT (IU/L)	1.5 ± 0.70	1.2 ± 0.66	1.7 ± 0.53	1.3 ± 0.57

ALP, alkaline phosphatase; AST, aspartate aminotransferase; ALT, alkaline phosphatase; BUN, blood urea nitrogen.

Values are presented as mean ± SD.

^*^ and ^**^ indicate significant difference at *p* < 0.05 and *p* < 0.01 levels, respectively, when compared with the vehicle control group.

**Table 5:** **Urinalyses of animals treated with OJS for 4 weeks**

**Group**	**Volume (mL)**	**Glucose** ^**a**^	**Bilirubin** ^**a**^	**Ketone body** ^**a**^	**Specific gravity**	**Occult blood** ^**a**^	**pH**	**Urobilinogen (E.U./dL)**	**Color** ^**b**^
**Male**									
V. CONTROL	22 ± 4.5	0/5	0/5	0/5	≤1.005 ~ 1.020	0/5	7.0	0.2	S(5)
500 mg/kg	26 ± 12.4	0/5	0/5	0/5	≤1.005 ~ 1.020	0/5	7.0	0.2	S(5)
1000 mg/kg	13 ± 2.9	0/5	0/5	0/5	1.015 ~ 1.025	0/5	6.9	0.2	S(5)
2000 mg/kg	22 ± 11.6	0/5	0/5	1/5	1.010 ~ 1.025	0/5	6.8	0.2	S(5)
**Female**									
V. CONTROL	9 ± 2.0	0/5	0/5	0/5	1.015 ~ 1.025	0/5	6.5	0.2	S(5)
500 mg/kg	9 ± 3.8	0/5	0/5	0/5	1.010 ~ 1.025	0/5	6.8	0.2	S(5)
1000 mg/kg	9 ± 4.5	0/5	1/5	0/5	1.015 ~ 1.025	0/5	6.6	0.2	S(5)
2000 mg/kg	10 ± 4.8	0/5	1/5	0/5	1.010 ~ ≥1.030	0/5	6.5	0.2	S(4), Y(1)

^a^Number of animals with sign/Total number of animals observed.

^b^S, strawberry and Y, yellow.

### Cytotoxicity of OJS extract

We further tested the cytotoxicity of OJS against 23 different cell lines. Viability of the cells was evaluated by MTT assay. Cells were treated with various concentrations (0, 10, 20, 50, 100, or 200 μg/ml) of OJS extract for 24 h. As shown in Table 5, OJS had no significant effect on the viability of cells including SH-SY5Y, SK-N-SH (neuroblastoma), U-373 MG, U-87 MG (glioblastoma), HepG2, Hep3B (hepatocarcinoma), Clone M-3, B16F10 (melanoma), HEK-293, NRK52 (kidney cells), 3T3-L1, NIH-3T3 (fibroblast), HIT-T15 (pancreatic cells), HL-60, RBL-1 (leukemia), HT-29 (colon cancer), MCF-7 (breast cancer), HaCaT (keratinocytes), PC12 (phechromocytoma), LNCaP (prostate cancer), BEAS-2B (bronchial epithelial cells), and AGS (gastric adenocarcinoma) up to 200 μg/ml in all tested cell lines (Table 6). Data are consistent with the results of 4 week-repeated toxicity test in animal model.

**Table 6:** **Cytotoxicity of OJS in various cell lines**

**Cell line**	**Origin**	**Concentration (μg/ml)**					
		**0**	**10**	**20**	**50**	**100**	**200**
SH-SY5Y	Human, Neuroblastoma	100 ± 1.97	100 ± 3.48	99 ± 1.74	99 ± 3.34	98 ± 2.65	96 ± 1.55
Clone M-3	Mouse, Melanoma	100 ± 4.22	101 ± 5.59	98 ± 3.67	100 ± 8.26	99 ± 6.17	99 ± 10.22
SK-N-SH	Human, Neuroblastoma (brain)	100 ± 2.20	105 ± 2.71	104 ± 1.66	107 ± 3.00	105 ± 1.38	108 ± 3.09
U-373 MG	Human, Glioblastoma (brain)	100 ± 2.63	100 ± 4.28	97 ± 1.83	95 ± 1.44	96 ± 1.67	96 ± 4.22
U-87 MG	Human, Glioblastoma (brain)	100 ± 7.07	100 ± 2.56	96 ± 1.22	99 ± 4.13	100 ± 3.38	100 ± 4.74
HEK-293	Human, Kidney	100 ± 9.45	101 ± 3.50	109 ± 4.62	106 ± 3.60	96 ± 2.86	97 ± 3.14
HepG2	Human, Hepatoblastoma	100 ± 2.67	92 ± 3.49	93 ± 3.05	92 ± 4.92	99 ± 2.06	87 ± 4.32
Hep3B	Human, Hepatocarcinoma	100 ± 1.95	100 ± 2.56	101 ± 2.15	104 ± 0.51	96 ± 3.47	93 ± 0.85
RAW 264.7	Mouse, Macrophage	100 ± 5.72	112 ± 9.54	121 ± 3.42	117 ± 3.59	115 ± 1.71	113 ± 1.56
3T3-L1	Mouse, Fibroblast (pre-adipocyte)	100 ± 2.60	105 ± 3.71	105 ± 2.67	110 ± 1.95	109 ± 3.04	113 ± 4.31
HIT-T15	Hamster, Pancreas	100 ± 2.83	95 ± 15.62	97 ± 13.85	97 ± 16.78	96 ± 8.35	95 ± 7.53
HL-60	Human, Leukemia, lymphoblast	100 ± 1.13	100 ± 1.25	100 ± 1.50	99 ± 1.43	100 ± 1.97	98 ± 1.99
HT-29	Human, Colon cancer cell	100 ± 2.78	102 ± 1.36	121 ± 2.55	99 ± 1.79	102 ± 3.45	104 ± 4.85
NIH-3T3	Mouse, Fibroblast	100 ± 6.28	102 ± 5.43	103 ± 5.32	102 ± 6.82	105 ± 9.97	104 ± 5.99
B16F10	Mouse, Melanoma	100 ± 8.87	91 ± 2.43	91 ± 5.50	94 ± 3.17	98 ± 6.68	111 ± 3.72
MCF-7	Human, Breast cancer	100 ± 4.84	114 ± 11.1	114 ± 0.44	114 ± 4.23	110 ± 8.76	110 ± 4.33
HaCaT	Human, Keratinocyte	100 ± 1.55	98 ± 1.67	98 ± 1.00	99 ± 3.21	97 ± 3.81	102 ± 2.99
PC12	Rat, Adrenal medulla, pheochromocytoma	100 ± 1.28	102 ± 1.71	96 ± 1.54	98 ± 2.94	98 ± 2.12	93 ± 3.20
AGS	Human, Gastric adenocarcinoma	100 ± 1.31	99 ± 1.94	100 ± 3.8	94 ± 10.36	96 ± 1.83	100 ± 1.80
BEAS-2B	Human, Bronchial epithelial, normal cell	100 ± 2.00	102 ± 0.86	101 ± 1.24	101 ± 0.65	104 ± 3.42	105 ± 2.68
LNCaP	Human, Prostate cancer	100 ± 9.14	101 ± 5.75	102 ± 8.88	101 ± 7.03	102 ± 12.51	98 ± 6.86
RBL-1	Rat, Basophilic leukemia	100 ± 5.97	102 ± 9.42	102 ± 6.60	102 ± 1.80	103 ± 2.12	102 ± 0.58
NRK52	Rat, Kidney	100 ± 1.19	100 ± 1.19	99 ± 2.18	102 ± 1.01	102 ± 3.99	106 ± 2.65

## Conclusions

We previously reported the safety of OJS in acute and sub-chronic toxicity as well as genotoxicity studies in rats. In addition, adverse effect of OJS has not been reported in clinical studies. Our current present study demonstrate that oral administration of Oriental herbal formula OJS causes leukopenia in female SD rats treated for 4 weeks. Thus, the no observed adverse effect level (NOAEL) values of OJS administration are determined to 2000 mg/kg/day in male and 500 mg/kg/day in female rats. Furthermore, no cytotoxic effect of OJS was found up to 200 μg/ml in 23 different cell lines. The cytotoxicity information of OJS can be used for *in vitro* biological studies. Further pharmacological studies will be required to find out the cause of WBC decrease by OJS in female rats before applying clinical studies in humans.
